# Psoriasis colocalized within preceding vitiligo patches: A rare case of Wolf’s isotopic response and literature review

**DOI:** 10.1016/j.jdcr.2026.06.030

**Published:** 2026-06-17

**Authors:** H. Mark Kenney, Francisco Tausk

**Affiliations:** Department of Dermatology, University of Rochester Medical Center, Rochester, New York

**Keywords:** cohabitation, colocalization, immunocompromised district, melanocytes, psoriasis, vitiligo, Wolf’s isotopic response

## Introduction

Coexistence of psoriasis and vitiligo has been well-described and recognized since as early as 1890.^1^ A recent meta-analysis reported that vitiligo patients have a 3.43 odds ratio of developing psoriasis, while those with psoriasis have a 2.29 odds ratio for vitiligo based on 10 case-control or cross-sectional studies.^2^ While often comorbid conditions, these reports typically represent a mixture of both discrete and colocalized cutaneous distribution of each condition; only in rare circumstances have there been reports of predominant or strict confinement of psoriatic plaques specifically within vitiligo patches.^3^ As highlighting the tendency for coexistence and evident potential for specific cohabitation of these conditions are critical to elucidate the interconnected pathophysiologic mechanisms, we provide a comprehensive review of the psoriasis-vitiligo colocalization phenomenon. We also add to the existing literature a case of new-onset psoriasis developing exclusively within preexisting areas of longstanding vitiligo and clarify the classification of this clinical finding as a rare example of Wolf’s isotopic response.

## Case presentation

**Fig 1:**
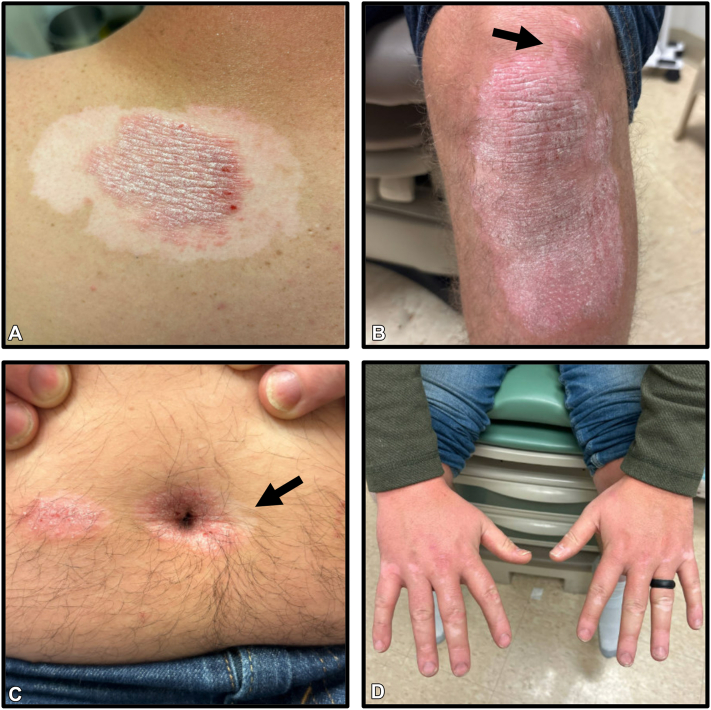
Psoriasis predominately colocalized within vitiligo patches. Erythematous plaques with silver scale consistent with psoriasis were found cohabitating the same cutaneous regions with preceding depigmented patches of vitiligo on the left upper back **(A)**, knees **(B)**, and periumbilical region **(C)**. Certain areas of vitiligo did not exhibit superimposed psoriasis, including several discrete patches overlying bilateral dorsal fingers **(D)**. *Black arrow* depicts edge of underlying vitiligo predominately covered by superimposed psoriatic plaques **(B** and **C)**.

**Fig 2:**
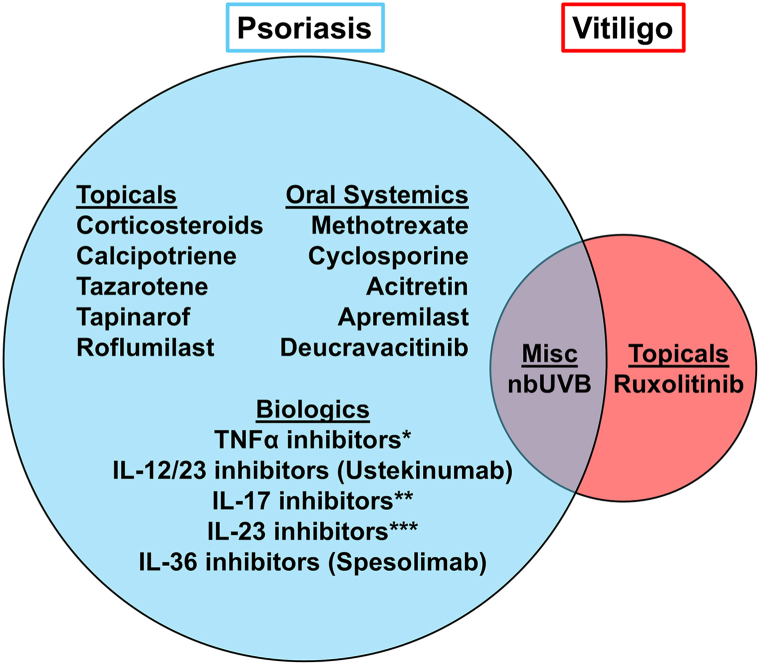
Psoriasis and vitiligo exhibit minimal dual-therapy options. A Venn diagram illustrates FDA approved treatment options for psoriasis (blue) and vitiligo (red) with narrow-band ultraviolet B (nbUVB) phototherapy (purple) as a potential first-line therapy for concurrent management of both conditions. ∗Tumor necrosis factor α (TNFα) inhibitors: etanercept, infliximab, adalimumab, certolizumab pegol; ∗∗Interleukin (IL)-17 inhibitors: secukinumab, ixekizumab, brodalumab, bimekizumab; ∗∗∗IL-23 inhibitors: guselkumab, tildrakizumab, risankizumab. *FDA*, Food and Drug Administration.

A 26-year-old male with history of scoliosis and childhood onset vitiligo presented to dermatology for management of plaque psoriasis that started at the age of 22 affecting the left upper back, periumbilical region, bilateral knees, scrotum, and buttocks. On presentation, the patient had erythematous plaques with overlying silver scale consistent with prior diagnosis of psoriasis vulgaris, but with a particular colocalization pattern directly within depigmented patches associated with longstanding vitiligo that started at age 6 (Fig 1, *A*). The patient confirmed the development of psoriasis predominately in areas where vitiligo had been present, including the scrotum, buttocks, bilateral knees, and periumbilical region, where associated depigmented patches are evident along the edge of the overlying psoriatic plaques (Fig 1, *B* and *C*; arrows). However, the patient also exhibited depigmented patches across the bilateral dorsal fingers without associated psoriasis (Fig 1, *D*). The patient had failed topical management with corticosteroids and calcineurin inhibitors, thus was initiated on methotrexate with consideration of guselkumab pending clinical course. For the vitiligo, the patient was advised to start topical steroids and calcineurin inhibitors with potential for utilization of topical ruxolitinib, depending on treatment response. Current treatment considerations for both psoriasis and vitiligo are provided in Fig 2.

## Discussion

The first definitive case of psoriasis confined to regions of vitiligo was reported in 1955 where psoriasis developed on an upper extremity affected by syringomyelia followed by segmental distribution of vitiligo on the same extremity, but all subsequent psoriatic plaques remained within vitiliginous skin.^4^ De Moragas & Winkelmann followed suit in 1970 with 2 similar reported cases of patients with longstanding psoriasis and later onset of vitiligo. Interestingly, both cases highlight that after the onset of vitiligo, once the psoriasis was effectively treated on the pigmented skin, psoriasis flares thereafter only maintained localization within the depigmented vitiligo patches.^1^ Despite the initial cases suggesting a paradigm of colocalization occurring in a psoriasis to vitiligo progression, the remaining 10 cases reported thereafter instead depicted the opposite situation with psoriasis developing superimposed on preexisting vitiligo,^5^, ^6^, ^7^, ^8^, ^9^, ^10^, ^11^, ^12^, ^13^ similar to the case presented here. Table I summarizes the confirmed cases of psoriasis and vitiligo with nearly or completely confined colocalization supported by images and case descriptions, Table II provides details on clinical features, and Table III highlights the rare frequency of this phenomenon. While a limited number of additional strict psoriasis-vitiligo cohabitation cases have been described in the literature from larger retrospective studies,^3^^,^^15^, ^16^, ^17^ these were omitted from the comprehensive summary in Table I given lack of details or images provided for a majority of cases to confirm the reported findings. In fact, Sharquie et al indicated 6 subjects with psoriasis confined to vitiligo lesions,^15^ but the example images provided could represent leading edge dermatitis related to active inflammatory vitiligo and not definitively psoriasis.

**Table I:** Previously documented cases of nearly or completely confined colocalization of psoriasis within vitiligo

Reference (y)	Sex	Age at presentation (y)	Onset of psoriasis (y)	Onset of vitiligo (y)	Distribution of colocalization
Selenyi (1955)^4^^,^∗	Male	?	45	48	Upper extremity
De Moragas & Winkelmann (1970)^1^	Male	57	∼30	∼50	Trunk, extremities
De Moragas & Winkelmann (1970)^1^	Male	53	41	50	Trunk, extremities
Papadavid et al (1996)^5^	Female	65	64-65	62	Trunk, extremities
Hwang et al (1998)^6^	Male	44	44-45	36	Trunk, extremities
De Sica & Wakelin (2004)^7^	Male	21	21	21	Trunk, elbows, scalp
Berger et al (2006)^8^	Female	32	16	“Before school age”	Trunk, lower extremities, neck
Berger et al (2006)^8^	?	45	>36	36	Trunk, lower extremity
Ujiie et al (2006)^9^	Male	53	47	38	Trunk, upper extremities
Dhar & Malakar (2009)^10^	Male	9	8-9	8	Lower extremities
Sharma et al (2022)^11^	Female	60	55-56	“Long-standing”	Trunk, neck, upper extremities
Gupta et al (2023)^12^	Female	62	61-62	∼40	Trunk
Royal et al (2023)^13^	Male	22	21-22	12	Trunk, upper extremities

Cases with available descriptions and/or images demonstrating psoriasis colocalized within vitiliginous skin are provided in chronological order of publication along with details including patient sex, age of case presentation, onset of psoriasis, onset of vitiligo, and cutaneous distribution of colocalized involvement.

∗ Summary derived from Koransky & Roenigk.^14^

**Table II:** Clinical features of reported psoriasis and vitiligo strict colocalization cases

Clinical feature	# of patients (%)
Sex	
Male	9 (64.3%)
Female	4 (28.6%)
Unknown	1 (7.1%)
Age	
Psoriasis onset (<18 y)	2 (14.3%)
Psoriasis onset (>18 y)	12 (85.7%)
Vitiligo onset (<18 y)	3 (21.4%)
Vitiligo onset (>18 y)	10 (71.4%)
Vitiligo onset (unknown)	1 (7.1%)
Relative disease onset	
Psoriasis before vitiligo	3 (21.4%)
Vitiligo before psoriasis	10 (71.4%)
Psoriasis concurrent with vitiligo	1 (7.1%)
Location∗	
Head and neck	3 (21.4%)
Trunk	12 (85.7%)
Extremities	13 (92.9%)

The combination of published cases (Table I) and the case presented here with confined colocalization of psoriasis and vitiligo (*n* = 14 patients) are summarized by clinical features, including sex, age, relative timing of disease onset, and location of cutaneous features. Underline provided to emphasize the temporal relationship between each condition.

∗ Sample size and percentages are more than total as many patients had involvement of multiple sites.

**Table III:** Diagnostic frequency of psoriasis, vitiligo, and concurrent conditions

Diagnosis (ICD-10 code)	Number of patients
Psoriasis only (L40; without vitiligo)	27,430
Vitiligo (L80; without psoriasis)	2950
Psoriasis and vitiligo (L40 and L80)	180

To further explore the frequency of the psoriasis-vitiligo association, we queried TriNetX at the University of Rochester (2,318,918 patients with data available from March 2011 to May 2026; no patient-level data was obtained). As this is the only known case at our institution with strict psoriasis-vitiligo colocalization, it computes a 0.56% (1/180) frequency of those with both psoriasis and vitiligo. However, additional retrospective investigation is necessary to characterize the clinical features associated with discrete vs colocalized cutaneous involvement. Underline provided to emphasize the concurrence of each condition. *ICD-10*, International Classification of Diseases Tenth Revision.

### Psoriasis-vitiligo colocalization classified as Wolf’s isotopic response

• The psoriasis-vitiligo colocalization represents an example of an isotopic response, first coined by Wolf et al in 1995, to characterize the onset of a new skin disorder occurring at the exact same site as an unrelated skin condition.^18^
• Wolf’s isotopic response (WIR) was described in contrast to the well-known Köbner isomorphic response where new lesions of a preexisting skin condition occur at sites of injury.^19^
• The concept of WIR was developed in the setting of postherpetic cutaneous responses, where several new-onset diseases have been frequently documented as localized to prior affected areas of herpes zoster.^20^ A subsequent update over 15 years later continued to reference WIR predominately occurring within preceding herpes zoster followed by other herpes infections, including herpes simplex.^21^
• More recently, WIR and other related phenomena has been enveloped into a more generalized conceptualization of the “immunocompromised district” with the goal to understand the underlying processes that predispose particular cutaneous regions to susceptibility of disease onset.^19^

### Potential shared pathophysiologic mechanisms of psoriasis and vitiligo

• Vitiligo evidently creates an “immunocompromised district” with risk of psoriasis development in particular patients; this further highlights the potential role of melanocytes is psoriasis pathogenesis.
• Since the description of circumferential hypopigmented zones occurring around resolving psoriasis plaques by Woronoff in 1926 (Woronoff ring), further investigation has revealed that related inflammatory cytokines (interleukin-17 and tumor necrosis factor-alpha) suppress melanin synthesis.^22^
• Arakawa et al identified a psoriatic infiltrative CD8^+^ T-cell clone that responded to melanocyte-specific antigens.^23^
• Other studies suggest direct cytotoxic effects of the psoriatic type 1 inflammatory milieu on melanocyte homeostasis by disrupting E-cadherins via matrix metalloproteinases leading to melanocyte detachment and apoptosis with progression to vitiligo.^24^
• Prior cases of vitiligo preceding psoriasis highlight the anti-melanocytic T-cell infiltrate producing T_H_1-associated cytokines such as tumor necrosis factor-alpha proposed to promote psoriasis onset.^8^
• Persistence of colocalization may function through cohabitation of disease-related tissue resident memory T-cells that regulate continued concomitant recurrence and/or dual-response to trauma with koebnerization.
• The unique psoriasis-vitiligo colocalization raises several questions warranting further investigation, including the potential pathways for anti-melanocytic autoinflammatory T-cells to regulate psoriasis proliferation and possible melanocyte-driven mechanisms reducing the likelihood of psoriasis onset in pigmented skin.

## Conflicts of interest

None disclosed.
